# Measurement Matters: Measuring Engaged Research in the Real World

**DOI:** 10.1093/geroni/igaf122.1276

**Published:** 2025-12-31

**Authors:** Erin McGaffigan, Marc Cohen, Tam Nguyen, Airia Papadopoulos, Myrna Finn, Melissa Destrampe, Sophia Webber, Alexa Fleet

**Affiliations:** Collective Insight, North Reading, Massachusetts, United States; University of Massachusetts Boston, Boston, Massachusetts, United States; Boston College William F. Connell School of Nursing, Chestnut Hill, Massachusetts, United States; Collective Insight, North Reading, Massachusetts, United States; Osher Lifelong Learning Institute, Pittsfield, Massachusetts, United States; Collective Insight, North Reading, Massachusetts, United States; Collective Insight, North Reading, Massachusetts, United States; University of Massachusetts Boston, Boston, Massachusetts, United States

## Abstract

Engaging older adults and individuals with chronic conditions to drive research design, implementation, and dissemination is gaining increased attention. Many researchers lack clarity on how to operationalize engagement frameworks, creating frustrations among those who try and within the communities they seek to engage. To address this gap, the Measurement Matters project, funded by a Patient Centered Outcomes Research Institute (PCORI) Research Award (SOE-2022C2-28570), created and tested the PCOR-EM, a comprehensive tool to measure engagement activities and their potential impact on research outcomes. Collective Insight, LeadingAge LTSS Center @UMass Boston, Boston College Connell School of Nursing, in partnership with our Technical Advisors, Steering Committee, and Pilot Partner Subcommittee, developed the PCOR-EM through expert consensus methods, focus groups, and cognitive testing in 2024. In 2025, we tested the PCOR-EM with approximately 250 engaged research projects from the National Patient-Centered Clinical Research Network (PCORnet) – a network of diverse healthcare institutions across the U.S. Through this presentation, we will share the methods used and findings formulated from this multi-phase process, including our revised conceptual definition of engagement and key domains important for measurement as described by engaged researchers, engagement facilitators, and patient partners themselves. We will share the piloted PCOR-EM core concepts and discuss preliminary findings from our pilot testing with engaged research projects. These findings, as well as lessons learned on the use of advisory structures inclusive of patients and community partners, will provide a foundation for operationalizing key domains to support and evaluate researchers’ meaningful community engagement practices.

